# Exploring medical students’ perspectives of physician leadership

**DOI:** 10.1186/s12909-022-03971-x

**Published:** 2023-01-05

**Authors:** Albert Vo, Jacqueline Torti, Wael Haddara, Nabil Sultan

**Affiliations:** 1 Family Medicine, Western University, London, ON Canada; 2 Centre for Education Research & Innovation, Western University, London, ON Canada; 3 Critical Care Medicine, London Health Sciences Centre, London, ON Canada; 4 Division of Nephrology, London Health Sciences Centre, London, ON Canada

**Keywords:** Physician, Leadership, Character, Competence, Commitment, Clerkship

## Abstract

**Background:**

Leadership has been recognized as an important competency in medicine. Nevertheless, leadership curricula for Canadian medical students lacks standardization and may not be informed by medical students’ perspectives of physician leadership. The purpose of this study was to elicit these perspectives on physician leadership.

**Methods:**

The present study utilized semi-structured interviews to ascertain the views of medical student participants, including students in their first, second and third years of medical school, on physician leadership. Interview questions were based on ‘the 3-C model’ of physician leadership, which includes three aspects of leadership, namely character, competence and commitment. The interviews were audio-recorded, transcribed and then coded using thematic analysis.

**Results:**

The medical students of this study provided rich examples of resident and staff physicians demonstrating effective and ineffective leadership. The participants identified the importance of character to effective physician leadership, but some participants also described a feeling of disconnect with the relevance of character at their stage of training. When discussing physician competence, medical students described the importance of both medical expertise and transferable skills. Lastly, the leadership aspect of commitment was identified as being relevant, but medical students cautioned against the potential for physician burnout. The medical student participants’ suggestions for improved leadership development included increased experiences with examples of physician leadership, opportunities to engage in leadership and participation in reflection exercises.

**Conclusions:**

Overall, the study participants demonstrated an appreciation for three aspects of leadership; character, competence and commitment. Furthermore, they also provided recommendations for the future design of medical leadership curricula.

**Supplementary Information:**

The online version contains supplementary material available at 10.1186/s12909-022-03971-x.

## Background

Leadership is an essential competency for physicians engaged in clinical practice, and it manifests in leading clinical teams effectively, educating junior trainees, interacting with patients and family members, and participating in quality improvement initiatives [1–3]. The Royal College of Physicians and Surgeons of Canada reinforced the importance of physician leadership by replacing the CanMEDS’ ‘manager role’ with the new ‘leader role’ in 2015 [4]. In light of this need for physicians to demonstrate leadership, it is imperative that leadership be similarly emphasized in medical education. Unfortunately, leadership has not always been prioritized in medical curricula. Reasons for this disconnect include medical students not being provided with adequate support to exercise leadership in hospital settings, a lack of funds to implement leadership curricula, and time constraints in the medical school curriculum [5–7]. Other key barriers include a lack of awareness by medical students of leadership as a competency and the perception that there are multiple competing competencies to be learned [5].

Despite these challenges, it is encouraging that several medical schools have introduced leadership curricula [6, 8, 9]. Despite these early efforts, two important considerations remain relatively unaddressed. First, there is a lack of standardization of leadership curricula between Canadian medical schools. Second, incorporating the perspectives of medical students is likely to produce more effective curricula [10]. Medical student curriculum representatives have been shown to be able to minimize content overlap between courses and to aggregate the opinions and criticisms of their classmates [11]. Nevertheless, to date, there are few qualitative studies that specifically address medical students’ perspectives on medical leadership.

The current medical education leadership literature lacks a robust understanding of medical students’ perspectives on leadership. In addition, there is often a lack of grounding leadership development curricula in established leadership frameworks [12]. One such framework is the 3-C leadership model, a popularized model of leadership development used both inside and outside of medicine, yet there is no available literature on the perspectives of medical students on this framework. We aimed to address this gap through our research question: what are the perceptions of medical students on physician leadership, through the prism of the 3-C framework of leadership? This study will provide important insights for educators and administrators to help optimize the design and implementation of leadership education targeting medical students and to recognize how the 3-C framework resonates with medical students. This study will provide insights into the transferability of a pre-existing model of leadership to undergraduate medical education and can help inform standardized approaches to leadership education and training in undergraduate medical education.

## Methods

The purpose of this study was to explore the perceptions, attitudes, and ideas of medical students towards physician leadership. A descriptive qualitative study design was chosen to elicit medical students’ perceptions on physician leadership [13]. This process was facilitated by introducing study participants to a framework of leadership used in the field of business. The business field was deemed an appropriate reference point as many physicians have enhanced their leadership skills through training in business programs such as the Master of Business Administration (MBA) [14, 15]. The specific framework introduced to medical students in this study was ‘the 3-C model,’ named after three fundamental components of leadership: Character, Competence and Commitment [16]. Prior to being asked about their perspectives on these aspects of leadership, participants were provided with the study definitions of each of the three components of the 3-C model. Character was defined as, “Internal traits, virtues and values of an individual such as integrity, courage, justice, compassion and humility. This aspect of leadership manifests itself when these personal values of an individual are consistently applied in different environments and situational pressures.” Competence was defined as, “necessary knowledge/skills, social skills, strategic skills, etc.” Commitment was defined as, “the hard work and motivation for developing leadership, which may involve positively engaging one’s team or making sacrifices for the greater good.” The 3-C framework of leadership is taught to MBA students in a primarily theoretical setting rather than the real world. This approximates the setting of most medical students in pre-clerkship, i.e., the first two years of their medical training. Specifically, the character element of leadership has been taught to business students through an MBA elective course which features learning modalities such as mentorship, journaling, and workshops designed by participants [17]. The character component of leadership has been further subdivided into eleven dimensions, namely: transcendence, drive, collaboration, humanity, humility, integrity, temperance, justice, accountability, courage, and judgement [18]. In addition, in this study, a distinction was made between two forms of leadership, i.e., positional, and dispositional leadership [19]. While positional leadership focusses on the titles and status of an individual as a leader, dispositional leadership emphasizes the traits and characteristics a leader demonstrates. The study was primarily designed to focus on the dispositional model of leadership.

### Recruitment

Medical students in their first three years of training were recruited from the Schulich School of Medicine & Dentistry to participate in the study. Purposeful sampling was used to recruit both pre-clerks (first 2 years of training) and clerks (third year of training), who would be able to provide unique perspectives on physician leadership as a result of being at different levels of training. First and second year medical students were recruited via email. A single email invitation requesting participation in the study was sent to all first- and second-year students. Every student who responded to the email was offered an interview. Participant recruitment took place over two years. Third year clerks who had completed or were in the process of completing their internal medicine rotations were also contacted via email. In addition, clerks who had completed their surgical rotations were contacted. Recruitment focused on these specific medical rotations as the researchers wanted to elicit medical students’ perceptions of physician leadership in team-based clinical settings. A.V. also performed in-person recruitment of 3rd year medical students on the clinical teaching unit (CTU) of Internal Medicine.

### Data collection

Participants were invited to take part in an hour long semi-structured interview. The interviews were one-on-one sessions between the interviewer and a single participant. All interviews were audio-recorded. Participants signed a consent form agreeing to participate in the study and have their interview audio-recorded, with their understanding that their de-identified quotes may be included in publications arising from this work. These interviews included questions on effective and ineffective leadership as well as the 3Cs of leadership; character, competence and commitment [16]. The study interview guide was designed by N.S. based on recent literature from the field of leadership. This interview guide has also been used in a previous study [20]. To adapt the guide to the present study, it was pilot tested with a medical student researcher at the Centre for Education Research and Innovation. Interviews were conducted until the point of theoretical sufficiency [21].

The interviews were facilitated by A.V., who at the time of data collection was a first-year medical student. A.V. was attending the same medical school as the participants of this study. This interviewer was selected as he had a period of prolonged engagement with the participants of the study [22]. The majority of interviews took place in the office space of the Centre for Education Research & Innovation at Western University. For the convenience of certain participants, interviews were also conducted either virtually or at University Hospital, London Health Sciences Centre.

### Data analysis

The audio recordings of the interviews were sent to a professional transcription agency. Transcribed interviews were then analyzed by A.V. through the process of thematic analysis [23]. This process included coding the transcripts within separate Microsoft Word documents. The codes were used to form a code book to ensure consistent coding [24]. The study themes were then derived from these codes. Key participant quotations, which were representative of each theme, were included in this study. Analysis was both inductive and deductive, being informed by the 3-C model of leadership [25]. The aim of our analysis was to capture the general perceptions of medical students on leadership and to assess to what extent these perceptions aligned with the 3-C model of leadership. Analysis meetings and debriefing sessions were held during the data analysis process between A.V., J.T. and N.S. to discuss key codes and themes. During these meetings, the researchers discussed ways to clarify and consolidate codes. Revisions to codes and themes were agreed upon by all researchers before proceeding.

## Results

Twenty-four medical students participated in individual interviews, of whom thirteen were female, thirteen were first-year medical students, four in second year and seven in third year. As the participants of this study comprised students in their pre-clerkship and clerkship years, their perceptions of physician leadership varied accordingly. One reason for this variance was that clerks often directly interacted with physicians in a clinical environment, whereas pre-clerks predominantly interacted with physicians in other settings such as lectures, small group sessions, research projects and observerships, i.e., short-term clinical shadowing opportunities. Accordingly, medical students who worked more closely with physicians were able to provide more detailed examples of effective and ineffective physician leadership.

Overall, the medical students in this study believed that character, competence and commitment were important aspects of physician leadership. Participants provided examples of behaviours demonstrated by physicians who were proficient in these areas of leadership as well as by physicians who were lacking in these areas. The participants also elaborated on how key stakeholders such as medical learners, healthcare team members and patients benefited from physicians who demonstrated these leadership qualities.

### The relevance of character

#### Importance of character

In this study, character was discussed as an overall quality of leadership as well as in terms of its separate dimensions. As an overall quality of leadership, medical students identified that character was necessary to establish trust and respect with team members. Character was also seen as being important for patient interactions, clinical decision making, and when physicians were faced with ethical scenarios. As one medical student stated:

“To work as a physician, to be in these stressful situations, to give of yourself day after day, you have to have a strong moral and ethical centre that places paramount on acting for the benefit of others. The principles that you hold shape how you act so you have to have principles of compassion, of humility, of honesty, and of determination…” (Participant 13, Pre-clerk)

#### Lack of character

In contrast, poor character was suggested to have a negative influence on team culture and communication. Participants described the negative results of leaders demonstrating poor character:

“I would say it’s [character is] extremely important, because if a person doesn’t have good character, they would not be trusted as a leader. And even if they’re a leader more along the lines of a dictator rather than a true leader, having poor character essentially means that you’re not even fit to be physician, so not necessarily a physician leader.” (Participant 22, Clerk)

Despite this, some study participants, particularly students in their pre-clerkship years, stated that they did not find character particularly relevant at their stage of training. These students often felt that, in medical school, acquiring medical knowledge and expertise was prioritized over character development:

“I feel like right now in our everyday experiences we are in our own little heads in our own little bubbles trying to learn as much as we can and not really working toward being a leader in any of these. They are hopefully there and will develop over time, but right now in medical school I don’t think any of them are relevant. Right now, we just have our heads in books, unfortunately.” (Participant 11, Pre-clerk)

#### Importance of specific character dimensions

Medical students described different clinical scenarios, which highlighted the importance of demonstrating various character dimensions. Character dimensions could be seen as benefiting the leader themselves, their clinical team, or their patients.

For example, participants expressed how leaders could personally benefit from demonstrating self-reflection and a desire to improve upon themselves, i.e., by demonstrating humility.

As a participant explained:

“I think one of the things that I’ve really seen and been impressed by is physicians that are able to say, I don’t know or that’s something I need to check back on…” (Participant 23, Pre-clerk)

Participants also described how it was important for clinical leaders to demonstrate character dimensions, such as collaboration and humanity, in team-based situations. Specifically, medical students explained how working with an approachable and open leader resulted in team members feeling comfortable as well as the team being productive overall. As one medical student described:

“As a clerk, it’s nice when someone actually does that because I often feel that I’m wasting people’s time, or this question I have isn’t one that I should be asking, I should know it already. When someone is so encouraging, and open to answering questions, it makes me feel more comfortable working on the team, and it makes the care better.” (Participant 19, Clerk)

Finally, some medical students recognized that integrity was important to patient care and an absence of this character dimension could result in consequences for patients.

One student described how she, personally, needed to demonstrate integrity in a difficult patient encounter to ensure appropriate care.

“And maybe some integrity, like it would have been easy to just let him [the patient] go home and suffer the potential consequences, but I tried my best to let him know that it would be best for him to stay in hospital to correct his DKA [diabetic ketoacidosis] before sending him back.” (Participant 20, Clerk)

#### Relationship between character and competence

Several medical students also drew connections between the leadership concepts of character and competence. Some participants explained how an individual’s character could motivate them to improve upon their competence. For example, it was argued that leaders with strong character would be able to self-reflect on personal areas of incompetence and be more open to improving these deficiencies. As one student stated:

“And, I think with character, as well, if you’re not competent at something you wouldn’t try to hide it, you’d be a lot more willing to improve or delegate the parts that aren’t competent. Whereas, if you have a bad character, even if you’re very competent, people might reach a point where they’re not competent in something and someone might try to hide that or end up with poor consequences.” (Participant 5, Pre-clerk)

Another student explained how character dimensions such as drive, accountability, and courage would help a leader to become competent. Other students discussed how aspects of a leader’s character, such as approachability, judgement and accountability could be influenced by their level of competence. As one of these students explained:

“In order to make good judgement, you need to be competent. If you want to work well with a team, you have to be competent as well, you have to understand your viewpoint and be able to share it as well. I guess competence also flows into accountability as well, you’ve got to be able to understand when you might be wrong and be able to accept that.” (Participant 15, Pre-clerk)

### The relevance of competence

#### Importance of competence – medical expertise

The participants of this study discussed two aspects of competence with regard to leadership. These components of competence included the medical expertise required to be a physician and the transferable skills, which physician leaders possess.

Medical expertise was considered important because it was a means of making appropriate medical decisions, gaining the respect of team members and physician colleagues, and acquiring leadership positions. Participants also identified several consequences that physicians face when they demonstrate a lack of medical expertise. These issues included compromised patient care, over-reliance on team members, acquiring a poor reputation, being unable to complete tasks, missing out on employment opportunities, and loss of patient and learner trust.

One student explained how it is important for physicians to have an understanding of the roles and responsibilities of their allied health professionals.

“But I think a physician has to be competent at that stuff…It’s things like drawing up vaccines out of the little things, or doing the catheter insertions or something. I think, even if I’m not going to be doing this day to day, it would be kind of embarrassing if I had to do it and couldn’t because then that would demonstrate a lack of competence.” (Participant 12)

#### Importance of competence – transferable skills

Regarding the transferable skills of physician leadership, participants discussed the importance of communication, organization and teaching ability. Although teaching is classified under the “Scholar” as opposed to “Leader” competency of the CanMEDs framework, aspects of teaching, i.e., constructive feedback and oral communication, have been described as transferable skills [26, 27]. Communication was seen as an important skill for eliciting patient concerns as well as for leading clinical teams. In clinical teams, communication was a means of providing the team with instructions, information, objectives, and policies. One participant described the effective and flexible communication skills of an emergency medicine resident, who he had observed.

“Through a lot of training, he fostered good group communication, but he didn’t just tell people how they were going to communicate, he fostered a consensus over how we should communicate, and he fostered a consensus over how he should communicate to the team and actually listened.” (Participant 2)

Participants provided a variety of reasons for why they considered organizational skills to be important including: managing various responsibilities such as research and patient care, guiding and leading clinical teams, and for accomplishing personal tasks. One participant described how her preceptor’s organized approach allowed him to be more time efficient.

“He was great at organization. Because he would come in at 8:00 something, he would already have a sheet, sort of a quick summary of each of the patients, so that if you’re a third-year student just walking in, never been to CTU, you weren’t going to get lost, he was helping you. The sheet would then help you get through the day…Which meant that then he would get through the day faster, and on time at the end of the day, with time for teaching. I felt like that was a very good learning experience.” (Participant 21, Clerk)

When discussing teaching methods, participants identified characteristics of an effective physician teacher. They valued physicians who demonstrated openness and motivation for instructing students. In addition, teachers who were able to tailor their instruction to the knowledge level of their students were considered effective. One student explained how effective physician leaders would also take into account the personal learning interests of their students:

“I find with the physicians, there are some that are very passionate about teaching, and you can tell that right away. They’ll ask you what your own interests are and what you want to do and what you’re comfortable with doing, to try to make it a good learning experience for you. That’s something that I really am happy with.” (Participant 14, Pre-clerk)

### The relevance of commitment

#### Relationship between competence and commitment

Throughout their interviews, some students also showed how the leadership concepts of competence and commitment were connected. For example, two students explained that a leader’s commitment could drive them to become more competent. Other students described situations where a physician’s commitment led to them being more knowledgeable and competent about their patients’ conditions. As one student explained:

“So, they stay on, like, they come in extra early and stay on extra late so that, 1) we [students] learn and, 2) they know what’s going on with the patients…I feel when they’re on CTU, during the weeks that they’re on, they’re 100% committed. We would not be able to function or be as organized without their help running through everything and realizing what the active issues are that we could have missed.” (Participant 18, Clerk)

#### Importance of commitment

The participants of this study provided key examples of residents and attendings demonstrating commitment to patient care and their clinical teams. Actions such as devoting time to teaching, reviewing patient lab work and results from home, and checking student dictations at the beginning of the day were seen as examples of behaviours, which demonstrated a physician’s commitment. One participant described how the commitment of one physician, who was willing to step in for an ill resident, helped the team.

“…one of the R2s was away, and the senior resident got sick. If the physician hadn’t been that committed either the senior resident would have had to stay while they were sick, or I could imagine the R1s and the med students just running the team…He basically just…he can’t do the whole team himself, but he really took on the role of the senior resident, and helped us more directly, and asked us more questions, and was more present with us, when they were away.” (Participant 19, Clerk)

Study participants also explained why they considered commitment to be important to physician leadership. One of the main reasons medical students believed that commitment was important was the need for physicians to lead by example. Teaching medical students was seen as one important way for physicians to demonstrate their commitment. When physicians are invested in their team’s education, learners may feel a greater sense of fulfillment with their daily duties, gain important skills, such as interpreting ECGs, and benefit from the experience and insight of medical experts.

“The best…at least from my perspective as a third-year student…the best consultants and residents are those that take, it doesn’t have to be a lot, but 10 to 20 min every day, pick a topic, and just go through it. Because we can read it and read it on our own forever, but we’ll never get the clinical pearls or the wisdom that these folks can distil in five or ten minutes.” (Participant 21, Clerk)

#### Lack of commitment

In contrast to these examples of positive actions, which demonstrated physician commitment, some participants also described behaviours, which represented a lack of physician commitment. Poor commitment could be shown by neglecting to provide students with feedback, relying on residents excessively, and not being available to speak with team members. Some of the perceived consequences of leaders not demonstrating commitment included team members feeling resentful and wishing to leave the team, becoming less motivated and choosing not to obey their leader. This lack of commitment was described by the following medical student.

“Some junior residents, for example, when they are surgery residents, then I see their commitment to the team. But when they’re an off-service resident on surgery, they are just kind of a more passive role where they do what they’re asked to do and no more than that” (Participant 22, Clerk)

#### Consequences of overcommitment

Although most participants recognized the value of commitment to physician leadership, some students also raised concerns about overcommitment and burnout. Aside from their clinical and teaching duties, physicians may also have commitments to their families, communities, and personal wellness and self-care.

“You really need someone who can balance both their work and their personal life. So, to me, that’s what sacrifice kind of brings up is the idea of a doctor who’s going to clinic from 9:00 to 5:00 and then is also running X, Y, and Z and never sees their family and never goes home on the weekend or never takes a vacation. And, I think that’s a little bit more outdated, because people do burn out and they do become less effective leaders.” (Participant 5, Pre-clerk)

### Leadership development

#### Effective methods of leadership development

Throughout their interviews, the medical students provided suggestions for how leadership could be incorporated into the medical curriculum. The participants also identified strategies which they considered to be ineffective for developing leadership. Participants’ recommendations for future leadership curricula can be summarized under three main categories. First, medical students emphasized the importance of having clinically relevant examples of leadership. Second, participants also recognized the importance of having opportunities where they could actively engage in leadership. Finally, medical students believed that reflecting on one’s experiences was an important method for developing leadership.

Participants offered various ways by which students could be exposed to examples of medical leadership. Their suggestions included providing students with physician mentors and role models demonstrating strong leadership and teaching abilities; encouraging physician lecturers to share their leadership experiences both in clinic as well as the community; and reviewing clinical cases that illustrated the importance of leadership skills.

“There should be maybe more examples of how physicians lead in their community because we find out a lot about physicians’ clinical aspects of their careers. But unless you spend time observing or you’re interacting with physicians for some other reason, you don’t find out about what they’re doing as leaders with their medical students, their residents, projects they might be doing and that kind of stuff.” (Participant 6, Pre-Clerk)

The participants also offered various innovative methods to directly engage medical students in leadership development. Some suggestions included providing medical students with a teaching role in clinical skills sessions; structuring research projects to give medical students a more prominent and independent role; as well as having medical students be involved in extracurricular activities such as clubs. Another student explained how actively practicing leadership would help medical students to demonstrate this skill in high pressure scenarios:

“I feel like if you just talk about leadership and you can take notes, but if you don’t have an opportunity to apply it, I don’t think that it will stick with people. Especially if it ends up being like a situation with some form of pressure…I feel like being put in applicable situations would be important for making sure that leadership is learned not only taught…” (Participant 3, Pre-clerk)

With regards to reflection, various participants described situations where they had thought back to times when they had displayed ideal or poor leadership. This reflective process allowed them to further develop themselves as leaders. Students described journaling or keeping a professional portfolio as one method for inducing reflection.

#### Ineffective methods of leadership development

Nevertheless, some participants expressed concerns and provided feedback regarding the way that new leadership curricula would be implemented. One concern was that presentations or workshops on leadership may not garner enough interest from students. Some participants also advised against teaching leadership in an overly standardized fashion. Having different approaches to leadership was considered important because all students have varying levels of interest in leadership. Similarly, it was argued that individuals can demonstrate leadership in a variety of ways. These differences in leadership methods were elaborated on by study participants.

“…but I don’t think there should be a ‘this is what leadership looks like class’ because leadership looks at a lot of things and giving it to students in a really defined way can sometimes narrow their minds to what leadership is and what leadership looks like.” (Participant 9, Pre-clerk)

## Discussion

The medical students in this study, regardless of their level of training, showed an appreciation for the importance of the ‘three Cs of leadership’. This finding is relevant as the 3 Cs model focuses primarily on the dispositional model of leadership, which posits that all individuals can demonstrate leadership [19]. As such, medical students can learn to demonstrate these aspects of leadership, which they have deemed to be valuable. Overall, character, competence, and commitment have been recognized as important in previous medical literature [28–30]. This is the first qualitative study to use semi-structured interviews of medical students to ascertain their perspectives on physician leadership. In addition, although the 3-C model has been described in the business field [31], this is the first study to formally introduce this model to medical students. Furthermore, the present study details specific suggestions for leadership development, which may be used by medical educators to design future effective curricula.

Many of the character dimensions in the leader character framework align with important principles and virtues in the field of medicine. In the present study, it was shown that the character aspect of leadership resonated with medical students. For example, participants in this study viewed humility as a means for leaders to improve upon themselves. Humility is necessary to understand the patient experience, to accept the input of other physicians, and to recognize the evolving nature of medical knowledge [32–34]. Furthermore, medical students in this study explained how the dimensions of collaboration and humanity could enhance team productivity and patient care. Collaboration has been valued as a means of improving information sharing between team members and of increasing the safety of patients [35]. Similarly, by practicing in a compassionate manner and demonstrating humanity, physicians may improve patient compliance and communication with their learners [36]. Integrity was another character dimension that was recognized as being important by study participants. It has been reported that by acting with integrity, and by behaving in a clear and consistent manner, physicians can garner the trust of others, a key requirement for effective leadership [37]. It has been argued that physicians can demonstrate integrity by only ordering appropriate medical investigations and by allocating an adequate amount of time for patient encounters [38]. Medical students are also expected to demonstrate integrity by abstaining from unprofessional behaviours such as academic cheating and by being transparent with patients who suffer adverse events [39].

A key theme that emerged in this study was the increased value placed on the attainment of medical knowledge over character development. One of the well-recognized challenges of undergraduate medical education is the amount of material that must be learnt in medical school [40, 41]. The finding that some medical student participants believed that character development should not be a primary concern, may be due to the medical “hidden curriculum,” i.e., aspects of the medical culture, which are learned without ever being explicitly expressed [42]. It has been suggested that a medical student’s empathy, an aspect of their character, can be adversely affected by the emphasis placed on medical knowledge by the formal and hidden curriculum [43]. Another study emphasized how the hidden curriculum could have a negative impact on another aspect of a medical student’s character, i.e., humility [44]. Medical knowledge is a well-recognized aspect of competence; however, the term competence in this study referred to not only medical knowledge, but also other leadership competencies such as social skills and strategic skills. Nevertheless, most participants focussed on the former, i.e., the medical expertise aspect of competence. This focus on medical expertise aligns with previous studies on the views of physicians and the public, which found academic competence to be a highly valued trait expected of physicians [45, 46]. Despite this emphasis on medical expertise, study participants still explored the relevance of other competencies of physician leadership, particularly communication, organization, and teaching ability. Effective communication has been valued in healthcare as a means of avoiding medical errors and facilitating transfer of information between physicians and their patients [26, 47]. The participants of the present study provided further evidence for the importance of physician communication, specifically in the context of clinical teams. Likewise, when surveyed elsewhere, medical students recognized organizational skills as an important soft skill for the field of medicine [48]. The participants of the current study provided reasoning for the importance of this skill including improving efficiency and for managing clinical teams. Lastly, participants in this study valued clinicians who demonstrated effective teaching skills, particularly those physicians who taught at a level appropriate for their learners, and this was perceived to be associated with effective physician leadership for students. In accordance with this finding, a study of medical students revealed that these learners appreciated clinician teachers whose feedback was appropriate for their skill level [27]. Conversely, a study of junior medical residents showed that they did not respond well to clinician teachers who created an impression that their students were less intelligent [49].

Commitment was also seen as a key aspect of leadership by the participants of this study. Traditionally, work commitment has been measured through the effort and time an individual devotes to their career [50]. Therefore, the medical student participants may have valued the commitment aspect of leadership due to the demanding nature of the field of medicine [51]. In one qualitative study, British general practitioners highlighted challenges such as increased patient complexity and unrealistic patient demands as contributors to their extensive workload [52]. In addition, as the medical students in this study were studying and working at an academic centre, many of the physicians with whom they interacted were involved in academic roles. In addition to their clinical roles, academic physicians have several other responsibilities such as attending conferences, sitting on committees, and educating learners [53]. The medical students of the present study appreciated the importance of physician commitment and its connection to impactful leadership. They provided a unique view that committed physicians help to promote learner education while ensuring the effective functioning of medical teams. The study participants highlighted that while it was important for physicians to be committed, they should be careful to avoid burnout. The concern for physician burnout has been well documented and contributing factors include excessive workload, overwhelming patient volumes, a lack of time to spend with loved ones, healthcare setting politics, and work being unacknowledged [54–56]. Overall, burnout is a multifactorial issue, which has been described as involving both personal and systemic issues. In addition, it has been argued that applying certain leadership styles, such as transformational leadership, may help to alleviate team members’ level of stress [57].

The present study demonstrated that medical students have an appreciation for, and were able to provide examples of the 3 Cs of leadership. The concepts of character, competence and commitment are not novel. Each of these aspects of leadership is implicit within the CanMEDS model for physicians [58]. Many of the dimensions of character, such as humanity, integrity and justice are contained within the Professionalism and Leader roles of CanMEDS. The commitment ‘C’ of leadership is likewise connected to the Professionalism role. Lastly, the competency aspect of leadership is encompassed by the Scholar and Medical Expert roles. One key difference between the 3 Cs of leadership and the 7 roles of CanMEDS is the clear interconnectivity between leadership concepts and highlighting the key roles character and commitment in particular play, rather than the common competency-centred approach. If Competency entails what a person “can do”, Commitment entails what a person “wants to do”, and Character entails what a person “will do” [16]. Therefore, each element of leadership interacts with one another to inform a leader’s actions. The participants of the current study further highlighted connections between each of the 3 Cs of leadership. For example, having strong character may motivate an individual to further develop areas where they lack competence. Similarly, physicians who demonstrate strong commitment to their patients inherently become more knowledgeable about and competent regarding their medical conditions. Therefore, several of the key expectations of physicians, as illustrated by the CanMEDS roles, can also be understood through the interconnectedness of the 3 Cs model of leadership.

The 3Cs of leadership have previously been applied to educational programs such as the MBA in the field of business. Now, with the suggestions provided by the current medical student participants, it may be possible to adapt this leadership model to medical education. A common theme amongst the proposed leadership educational methods included a focus on active learning as opposed to passive learning. The literature suggests benefits of taking an active learning approach such as improved memorization of material and having the opportunity to receive further explanation on topics [59, 60]. It is interesting to note that several of the active learning modalities proposed by the study participants already exist in medical education including mentorship [61], opportunities in extracurricular activities [62] and reflection [63]. Therefore, instead of proposing new medical leadership curricula, medical educators can consider leveraging and modifying these pre-existing learning opportunities. It is interesting to note that there are current studies of leadership curricula developed for medical students [8, 9]. However, medical student criticisms that arose in these leadership programs included certain program activities being viewed as non-value added and a lack of appreciation for the relevance of some of the concepts taught. The present study’s participants made recommendations, such as providing students with an individualized approach to learning leadership and avoiding overreliance on didactic teaching modalities like presentations and workshops, which may address some of these concerns.

### Limitations

There were a few limitations to this study. Although purposeful sampling was used to recruit both pre-clerk and clerk participants in this study, the majority of respondents were pre-clerks, with only seven participants being clerks [64]. This difference in participation rates may have been because the interviews were conducted during the summer months of July and August. Although this is a study-free period for pre-clerks, clerks continue to work during these months. In addition, all interviews were conducted at one medical school. Although this limits the generalizability of the study results, the transferability of the results was maintained [65, 66]. The results of this study are representative of the views of pre-clerks and clerks at one Canadian medical school, and this protocol could have similarly been applied to the other Canadian medical schools. Lastly, all study interviews were conducted by researcher A.V. The presumed advantage of this approach was that A.V. would have some degree of an “insider” status to the experiences of a medical student [67, 68]. However, there is a potential that some degree of objectivity may have been lost or some interviewee insights may have been assumed to be mutually understood as opposed to further explored. Although all interviews were conducted by A.V., this limitation was mitigated by having frequent study meetings to review relevant findings with researcher J.T, who was not directly involved in the medical school system.

One of the strengths of this study was the incorporation of the perspectives of both clerk and pre-clerks on physician leadership. There are several key differences between the clerkship and pre-clerkship environments, such as level of patient exposure and ability to perform medical procedures [69]. Therefore, it was valuable to be able to obtain perspectives from students at these different levels of training. In addition, another strength of the study was the semi-structured and iterative nature of the interviews, whereby the results of previous interviews informed future interviews [70, 71]. By employing this interview format, rich and novel data could be collected from the research participants to better understand their views of physician leadership.

### Next Steps

- 1. This study will assist medical educators in developing and reforming their leadership development efforts in undergraduate medical education. The study demonstrates that a popularized and well-explored leadership framework such as the 3-C model does indeed resonate with medical students and can provide a theoretical grounding for future leadership education in this stage of training. This study also demonstrated that medical students understand, appreciate and value the dimensions of Character & Commitment as they relate to leadership despite their lack of extensive clinical exposure. The results of this study should encourage educators to pursue a holistic approach to leadership development and resist the temptation of focussing on leadership competencies that may be easier to teach and evaluate. This will likely entail using non-didactic pedagogy such as mentorship, self-reflection and experiential learning, encouraging educators to be creative in their leadership curriculum-design.
- 2. Educators and researchers are encouraged to evaluate any attempts at providing the described holistic leadership development in undergraduate medical students to help inform future leadership development.
- 3. It would be valuable to determine medical students’ perceptions of physician leadership using clinical examples beyond surgery and internal medicine, as was the case in our study, and include other areas of medicine. This should include fields not centred around in-patient rounding such as family medicine, anesthesia or radiology.
- 4. A similar study can be repeated and expanded to include attending and resident physicians to explore if their perspectives similarly align with the medical students in this study.

## Conclusions

The findings of this study demonstrate that medical students recognize and value the importance of physician leadership. A popular framework of leadership development (3-C model) was found to resonate with the participants and with their general views on leadership. Students were able to appreciate the central roles character, competence and commitment play in supporting effective leadership. Students were able to provide examples of leaders who have either demonstrated proficiency or deficiency in each of these aspects of leadership. Furthermore, the study participants identified nuances in the different aspects of leadership, such as character being deprioritized at the pre-clerkship level of medical education or overcommitment leading to burnout. Some participants also provided ways in which each of the 3Cs of leadership were related to one another. In addition, the recommendations provided by the medical students in this study can be used by medical educators to help create effective, standardized leadership curricula in undergraduate medical education. Overall, the receptiveness of medical students to the 3Cs model of leadership demonstrated in this study provides medical educators with a robust rationale to utilize this framework in future leadership development curricula within undergraduate medical education. This means that leadership development needs to be approached holistically and with a balanced approach to develop effective future leaders.

## Supplementary Information

**Additional file 1.** Interview Guide.

## Data Availability

The datasets generated and/or analysed during the current study are unfortunately not publicly available to preserve participant privacy and confidentiality. Our ethics approval states that only the research team, the Health Research Ethics Board at Western University and the Lawson Quality Assurance and Education Program will have access to the study’s dataset. External access to the dataset requires an amendment to the ethics protocol and the London Health Sciences Centre (LHSC) Patient Experience Office can be contacted at 519–685-8500 ext. 52,036 to request this process. Data are however available from the authors upon reasonable request and with permission of the Health Research Ethics Board at Western University and the Lawson Quality Assurance and Education Program.

## References

[1] Chadi N (2009). Medical leadership: doctors at the helm of change. McGill J Med: MJM.

[2] Dhaliwal G, Sehgal NL (2014). Demystify leadership in order to cultivate it. Acad Med.

[3] Wilkie V (2012). Leadership and management for all doctors. Br J Gen Pract.

[4] Dath D, Chan M-K, Abbott C (2015). CanMEDS 2015: From Manager to Leader.

[5] Omar A, Shrestha A, Fernandes R, Shah A (2020). Perceived barriers to medical leadership training and methods to mitigate them in the undergraduate medical curriculum: A mixed-methods study of final-year medical students at two medical schools. Future Healthcare J.

[6] Richard K, Noujaim M, Thorndyke LE, Fischer MA (2019). Preparing medical students to be physician leaders: a leadership training program for students designed and led by students. MedEdPORTAL.

[7] Matthews JH, Morley GL, Crossley E, Bhanderi S (2018). Teaching leadership: the medical student society model. Clin Teach.

[8] Daaleman TP, Storrie M, Beck Dallaghan G, Smithson S, Gilliland KO, Byerley JS. Medical Student Leadership Development through a Business School Partnership Model: A Case Study and Implementation Strategy. J Med Educ Curric Dev. 2021;8:23821205211010480. 10.1177/23821205211010479 PMC8082997 33997287

[9] Dobson C, Cookson J, Allgar V, McKendree J (2008). Leadership training in the undergraduate medical curriculum. Educ Prim Care.

[10] Huppatz C (1996). The essential role of the student in curriculum planning. Med Educ.

[11] Milles LS, Hitzblech T, Drees S, Wurl W, Arends P, Peters H (2019). Student engagement in medical education: A mixed-method study on medical students as module co-directors in curriculum development. Med Teach.

[12] Sultan N, Torti J, Haddara W, Inayat A, Inayat H, Lingard L (2019). Leadership development in postgraduate medical education: a systematic review of the literature. Acad Med.

[13] Sandelowski M (2000). Whatever happened to qualitative description?. Res Nurs Health.

[14] Crites GE, Ebert JR, Schuster RJ (2008). Beyond the dual degree: Development of a five-year program in leadership for medical undergraduates. Acad Med.

[15] Collins-Nakai R (2006). Leadership in medicine. McGill J Med: MJM.

[16] Crossan M. Developing leadership character. Ivey Business Journal. 2012.

[17] Crossan M, Mazutis D, Seijts G, Gandz J (2013). Developing leadership character in business programs. Academy of Management Learning & Education.

[18] Crossan MM, Byrne A, Seijts GH, Reno M, Monzani L, Gandz J (2017). Toward a framework of leader character in organizations. J Manage Stud.

[19] Edwards J, Martin B, Alpert D, Greenberg K, Crilley K, Birdsall M, DiMura D (2016). Leadership. Schools that deliver.

[20] Torti JM, Inayat H, Inayat A, Lingard L, Haddara W, Sultan N. Perspectives on physician leadership: The role of character‐based leadership in medicine. Med Educ. 2022;56(12):1184–93. 10.1111/medu.14875 35818740

[21] Nelson J (2017). Using conceptual depth criteria: addressing the challenge of reaching saturation in qualitative research. Qual Res.

[22] Barusch A, Gringeri C, George M (2011). Rigor in qualitative social work research: A review of strategies used in published articles. Soc work res.

[23] Braun V, Clarke V (2006). Using thematic analysis in psychology. Qual Res Psychol.

[24] Roberts K, Dowell A, Nie JB (2019). Attempting rigour and replicability in thematic analysis of qualitative research data; a case study of codebook development. BMC Med Res Methodol.

[25] Armat MR, Assarroudi A, Rad M, Sharifi H, Heydari A. Inductive and deductive: Ambiguous labels in qualitative content analysis. The Quali Rep. 2018;23(1):219–21.

[26] Leonard M, Graham S, Bonacum D (2004). The human factor: the critical importance of effective teamwork and communication in providing safe care. BMJ Qual Saf.

[27] Goldie J, Dowie A, Goldie A, Cotton P, Morrison J (2015). What makes a good clinical student and teacher?. An exploratory study BMC medical education.

[28] Chestnut DH (2017). On the road to professionalism. Anesthesiology.

[29] Englander R, Carraccio C (2014). Domain of competence: medical knowledge. Acad Pediatr.

[30] Van Eaton EG, Horvath KD, Pellegrini CA (2005). Professionalism and the shift mentality: how to reconcile patient ownership with limited work hours. Arch Surg.

[31] Seijts G, Gandz J, Crossan M, Reno M. Character matters: Character dimensions’ impact on leader performance and outcomes. Organ Dyn. 2015;44(1):65–74.

[32] Chochinov HM (2010). Humility and the practice of medicine: tasting humble pie. CMAJ.

[33] Wadhwa A, Mahant S (2022). Humility in medical practice: a qualitative study of peer-nominated excellent clinicians. BMC Med Educ.

[34] Wu J, Lowenstein E (2020). Balancing confidence and humility in the diagnostic process. Diagnosis.

[35] Morley L, Cashell A (2017). Collaboration in health care. J med imag radiation sci.

[36] Nolan P. Time for humanity from doctors towards patients. Postgraduate Med J. 2003;79(938):667–8. PMC1742897 14707237

[37] Gunderman RB, Kane G (2012). Integrity and its role in professionalism. AJR Am J Roentgenol.

[38] Nayak D, Sarukkai S (2014). Integrity in medical practice. Indian J Med Ethics.

[39] Coverdale JH, Roberts LW, Balon R, Beresin EV, Louie AK, Guerrero AP, Brenner AM, McCullough LB (2016). Professional integrity and the role of medical students in professional self-regulation. Acad Psychiatry.

[40] Hill MR, Goicochea S, Merlo LJ (2018). In their own words: stressors facing medical students in the millennial generation. Med Educ Online.

[41] Klatt EC, Klatt CA (2011). How much is too much reading for medical students? Assigned reading and reading rates at one medical school. Acad Med.

[42] Hafferty FW. Beyond curriculum reform: confronting medicine’s hidden curriculum. Acad med: J Assoc American Med Colleges. 1998;73(4):403–7. 10.1097/00001888-199804000-00013 9580717

[43] Eikeland HL, Ørnes K, Finset A, Pedersen R. The physician’s role and empathy–a qualitative study of third year medical students. BMC medical education. 2014;14(1):1–8. 10.1186/1472-6920-14-165 PMC4128827 25108627

[44] Michalec B. The pursuit of medical knowledge and the potential consequences of the hidden curriculum. Health:. 2012 May;16(3):267–81. 10.1177/1363459311403951 21602249

[45] Dopelt K, Bachner YG, Urkin J, Yahav Z, Davidovitch N, Barach P. Perceptions of Practicing Physicians and Members of the Public on the Attributes of a “Good Doctor.” Healthcare (Basel). 2021;10(1):73. 10.3390/healthcare10010073 PMC8775310 35052237

[46] Leahy M, Cullen W, Bury G. “ What makes a good doctor?” A cross sectional survey of public opinion. Ir Med J. 2003;96(2):38–41. 12674150

[47] Chichirez CM, Purcărea VL (2018). Interpersonal communication in healthcare. J Med Life.

[48] Whittle SR, Eaton DG (2001). Attitudes towards transferable skills in medical undergraduates. Med Educ.

[49] Kisiel JB, Bundrick JB, Beckman TJ (2010). Resident physicians’ perspectives on effective outpatient teaching: a qualitative study. Adv Health Sci Educ.

[50] Morrow PC, McElroy JC (1986). On assessing measures of work commitment. J Occup Behav.

[51] Wallace JE, Lemaire J (2007). On physician well being—you’ll get by with a little help from your friends. Soc Sci Med.

[52] Croxson CH, Ashdown HF, Hobbs FR (2017). GPs’ perceptions of workload in England: a qualitative interview study. Br J Gen Pract.

[53] Steinmann AF, Dy NM, Kane GC, Kennedy JI, Silbiger S, Sharma N, Rifkin W (2009). The modern teaching physician—responsibilities and challenges: an APDIM white paper. Am J Med.

[54] Azam K, Khan A, Alam MT (2017). Causes and adverse impact of physician burnout: a systematic review. J Coll Physicians Surg Pak.

[55] Lee YY, Medford ARL, Halim AS (2015). Burnout in physicians. J R Coll Physicians Edinb.

[56] Collier R (2017). Physician burnout a major concern. CMAJ.

[57] Gill AS, Flaschner AB, Shachar M. Mitigating stress and burnout by implementing transformational‐leadership. Int J Contemp Hospitality Manage. 2006.

[58] Frank JR, Snell L, Sherbino J (2015). Can Meds 2015 Physician Competency Framework.

[59] Shoemaker EZ, Johnson C, Hilty DM, Fung CC (2022). Flipping a Single Lecture in a Survey Course to Active Learning: Do the Benefits Justify the Costs?. J technol behav sci.

[60] Kanthan R, Mills S (2005). Active learning strategies in undergraduate medical education of pathology: a Saskatoon experience. J Int Assoc Med Sci Educ (JIAMSE).

[61] Frei E, Stamm M, Buddeberg-Fischer B (2010). Mentoring programs for medical students-a review of the PubMed literature 2000–2008. BMC Med Educ.

[62] Cirone J, Saks NS (2015). Medical student engagement in extracurricular activities. Medical Science Educator.

[63] Winkel AF, Yingling S, Jones AA, Nicholson J (2017). Reflection as a learning tool in graduate medical education: a systematic review. J Grad Med Educ.

[64] Palinkas LA, Horwitz SM, Green CA, Wisdom JP, Duan N, Hoagwood K (2015). Purposeful sampling for qualitative data collection and analysis in mixed method implementation research. Adm Policy ment health ment health serv res.

[65] Shenton AK (2004). Strategies for ensuring trustworthiness in qualitative research projects. Educ Inf.

[66] Leung L (2015). Validity, reliability, and generalizability in qualitative research. J family Med Primary Care.

[67] Gair S (2012). Feeling their stories: Contemplating empathy, insider/outsider positionings, and enriching qualitative research. Qual Health Res.

[68] Bonner A, Tolhurst G (2002). Insider-outsider perspectives of participant observation. Nurs Res (through 2013).

[69] Mistry N, de Laplante S, Campbell CM. Assessing the Transition From Pre-Clerkship to Clerkship in a Four-Year Medical Program: Recommendations for Future Success.

[70] Saks M, Allsop J. Researching health : qualitative, quantitative and mixed methods / edited by Mike Saks and Judith Allsop. (2nd ed.). SAGE. 2013.

[71] Morse JM, Barrett M, Mayan M, Olson K, Spiers J. Verification strategies for establishing reliability and validity in qualitative research. Int J Qual Methods. 2002;1(2):13–22.

