# Supplementary material for: Exploring medical students’ perspectives of physician leadership
Source: BMC Med Educ. 2023 Jan 5;23:10. doi: 10.1186/s12909-022-03971-x (PMC9817360; doi:10.1186/s12909-022-03971-x)
Supplement: Supplementary file 1 — Additional file 1. Interview Guide. [file 12909_2022_3971_MOESM1_ESM.docx]

**Interview Guide**

**Exploring Medical Students’ Perceptions of Physician Leadership**

**Introduction**

- Introduce yourself and thank the participant for their time.
- Confirm that the participant can meet for approximately an hour.
- Ask if they have had a chance to read the consent form. Do they have questions? If they did not read it, offer to let them read it. If they waive their right, mention key points:
  - The interview will be audio-recorded but the transcript will be anonymized and any names mentioned will be deleted.
  - This research has no impact on your position in the hospital, university or training program.
  - Participation in the research study is voluntary and your information will be kept confidential. The principal investigator will be the data custodian. Your decision to participate or withdraw from the study will not impact your relationship with the training program, hospital department or division.
- Do you want to go over the form again? If you have any questions, feel free to ask me or contact my supervisors.
- Request their signature on 2 copies of the consent form: one for them to keep and one for our records.
- Start the audio-recording and say, “*I am interviewing you to understand your views and perceptions on the nature and importance of effective physician leadership. With this study, we hope to improve our understanding of what constitutes effective physician leadership and how we can develop physician leadership in medical trainees.*

The interviews will begin by giving a description of what is meant by the term ‘physician leadership’ for the purpose of our study. This will be done by reading the following statement:

- *Physician leadership may be understood in different ways. One way is to understand it as individuals who hold leadership positions (i.e. positional leadership) or as individuals who have the capacity to lead others in positive ways (we call this the disposition to lead). What we mean by physician leadership in this study is the ‘disposition to lead’ and not necessarily any positional leadership.*

Upon reading the statement above, the member of the research team will ask if this concept is clear in the participant’s mind and if any further explanation could be helpful.

**Background Questions**

1. Could you please tell me a little bit about where you are in your program?
2. Could you provide us with your own definition of what you think leadership is?
   1. Thank you very much for sharing
3. Have you had the opportunity to interact with other physicians (consultants, residents) during your schooling so far? What has that experience been like?
4. Has leadership come up in any of your education/schooling thus far? If so, please describe the circumstances.
   1. Do you believe that leadership should be incorporated into your education, and if so, how?

**Defining Effective/Ineffective Leaders**

1. What do you think makes an effective physician leader?
2. Do you have any examples of physicians that you think are effective leaders? Why do you think they are effective?
   1. Can you think of any other examples of effective physician leadership?
3. What do you think makes an ineffective physician leader?
4. Do you have any examples of physicians that you think are ineffective leaders? Why do you think they are ineffective?
   1. Can you think about any other examples of ineffective physician leadership?
5. You will soon be starting your clerkship. Describe the type of physician leadership you expect to see in your clinical rotations?
6. You have provided me with characteristics of effective and ineffective physician leadership, given that what would an ideal physician leader look like to you?
7. Do you see yourself being an effective physician leader one day? Why or why not?

**Leadership Characteristics**

1. Can you please rank each of the following characteristics and attributes on a scale from 1 (not very important) to 5 (very important) in association with physician leadership:

Organizational skills

Integrity

Sacrifice

Courage

Strategic thinking

Engagement

Financial competency

Drive

Aspiration

Follow up: Why did you rate these as higher/lower than the others? Do you have any feedback regarding the list?

**Character**

1. When referring to character we are referring to formation of internal traits, virtues and values of an individual such as integrity, courage, justice, compassion and humility. Character manifests itself when these personal values of an individual are consistently applied in different environments and situational pressures. Character can be described as ‘who one is’. How important do you think “character” is to effective physician leadership?
2. Can a physician be an effective leader without “character”?
3. Why do you think character is necessary/unnecessary to be an effective leader?

**Leader-Character Framework**

Have the participant review the leader-character framework before asking the next question.

A person’s Character manifests when that person experiences a situational pressure that requires them to exercise a specific character dimension, but the situational pressure makes it difficult to do. For example, a situational pressure in medical school may be working in a group with an uncooperative team member who strongly objects to the direction and opinions of the other team members. Useful dimensions here might be courage, honesty, and collaboration.

- How relevant do you think the leader-character framework is to your everyday experiences in medical school?
- Have you come across situational pressures in the classroom?

**Competence**

1. When referring to “competence" we are referring to having the necessary knowledge/skills, social skills, strategic skills, etc. A leader’s competence determines what they can accomplish. How important do you think “competence” is to effective physician leadership?
2. Can a physician be an effective leader without “competence”?
3. Why do you think competence is necessary/unnecessary to be an effective leader?

**Commitment**

1. When we refer to “commitment” we are referring to what one is willing to do. Commitment involves hard work and motivation for developing leadership. This may involve positively engaging one’s team or making sacrifices for the greater good. Commitment can be driven by having high aspirations for the goals of the team and organization. How important do you think “commitment” is to effective physician leadership?
2. Can a physician be an effective leader without “commitment”?
3. Why do you think commitment is necessary/unnecessary to be an effective leader?

**Closing**

1. Are there any additional comments you would like to make or was there anything else that you wanted to add that you didn’t get a chance to talk about?

Thank the participant for their time.

Close the interview by stating “*This is the end of the recording.*” and turn off the audio-recording.
